# Efficacy and safety of HD-MTX based systemic chemotherapy regimens: retrospective study of induction therapy for primary central nervous system lymphoma in Chinese

**DOI:** 10.1038/s41598-017-17359-1

**Published:** 2017-12-06

**Authors:** Xiao Han, Yali Ji, Mingqi Ouyang, Tienan Zhu, Daobin Zhou

**Affiliations:** Department of Haematology, Chinese Academy of Medical Science, Peking Union Medical College Hospital, Beijing, China

## Abstract

We performed a retrospective study of 49 patients with newly diagnosed primary central nervous system lymphoma (PCNSL), to compare the efficacy and safety of different high-dose methotrexate (HD-MTX) based systemic chemotherapy regimens as induction therapy. 25 patients received AB ± R alternative regimen (consist methotrexate, ifosfamide, vindesine, dexamethasone, carmustine and teniposide), while others received HD-MTX ± R regimen. The complete response rate and overall response rate of AB ± R group and HD-MTX ± R group were 36.83% vs. 33.33%, and 68.42% vs. 71.43%, while the 2-year OS and PFS rate were 71.43% vs. 74.62%, and 42.86% vs. 54.64%, respectively. In Age > 60 subgroup, the 2-year OS and PFS rate of AB ± R group and HD-MTX ± R group were 81.82% vs. 33.33%, and 54.55% vs. 33.33%. No significant differences were found in grade 3 or 4 toxicity rate. Generally, HD-MTX ± R regimen was not inferior to AB ± R alternative regimen, but AB ± R alternative regimen seemed achieving more survival benefits in the elderly. We suggest to adjust HD-MTX ± R regimen by changing the dose-reduction strategy especially in elderly patients and adding other powerful drugs that can well penetrate blood-brain barrier to improve the efficacy.

## Introduction

Primary central nervous system lymphoma (PCNSL) is a rare type of extra-nodal non-Hodgkin lymphoma, the incidence is increasing with aging, showing the highest incidental risk in patients over 75 year-old^1^. The lesions are often limited to the brain, leptomeninges, eyes, and spinal cord at diagnosis. The prognosis of PCNSL is considered poor. The prognostication of the disease is also distinct from the Ann Arbor index, the International Extranodal Lymphoma Study Group (IELSG) described 5 parameters associated with poor prognosis in PCNSL^2^, including age, ECOG score, LDH and CSF protein level, and tumor located in deep regions of brains. PCNSL management techniques include chemotherapy, whole brain radiotherapy (WBRT), high-dose chemotherapy supported by autologous stem cell transplantation (ASCT), surgery *et al*.^3^. High-dose methotrexate (HD-MTX) is considered the most important component in front-line PCNSL chemotherapy regimens^3^. However, the efficacy of HD-MTX monotherapy is not satisfying enough with the 2-year overall survival (OS) rate only 61–63%^4,5^. Given the evidence from multiple centers, HD-MTX based combination chemotherapy seems to be a superior option for induction therapy of PCNSL^6–8^. The constituent of combination chemotherapy remains a subject of some debate. In our research, we aimed to explore the efficacy and safety of different HD-MTX based systemic chemotherapy regimens. Also, considering the age as a significant parament for both incidence and prognosis, we paid special attention to the elderly population.

## Results

Forty-nine consecutive patients were enrolled in our retrospective analysis. 25 patients received AB ± R alternative regimen based therapy, while the other 24 patients received HD-MTX ± R regimen based therapy. 2 patients receiving AB ± R alternative regimen were lost to follow-up and excluded from the analysis in the AB ± R alternative regimen group, while no loss in the HD-MTX ± R regimen group. The median follow-up duration of patients received AB ± R alternative regimen and patients received HD-MTX ± R regimen was 37 months (range, 1–133 months) and 13 months (range, 1–52 months) respectively.

### Patient characteristics

The baseline characteristics and supplementary treatments of the patients are shown in Table 1. The median age at diagnosis of patients received AB ± R alternative regimen and patients received HD-MTX ± R regimen was 57 years (range, 17–78) and 56 years (range, 39–69) respectively. 15 (65%) patients in AB ± R alternative regimen group and 8 (33%) patients in HD-MTX ± R regimen group were male. Among the patients with detailed International Extranodal Lymphoma Study Group (IELSG) score, 57% in AB ± R alternative regimen group and 53% in HD-MTX ± R regimen group had higher IELSG score (3–4). Two patients in AB ± R alternative regimen group had leptomeningeal lymphoma, three patients in HD-MTX ± R regimen group had eyes involvement, and one patient in HD-MTX ± R regimen group had nerve root involvement.

**Table 1:** Baseline characteristics and supplementary treatments of patients with primary central nervous system lymphoma.

	AB ± R alternative regimen (n = 23)	HD-MTX ± R regimen (n = 24)	*p*-value
Age (years) *	57 (17, 78)	56 (39, 69)	
Age > 60	11/23 (48%)	7/24 (29%)	0.19
Gender, male	15/23 (65%)	8/24 (33%)	0.03
ECOG performance status > 1	17/22 (77%)	15/24 (63%)	0.35
LDH > 250U/L	6/18 (33%)	3/22 (14%)	0.26
CSF protein > 0.45 g/L	13/17 (76%)	13/17 (76%)	1.00
Deep lesion	9/21 (43%)	13/24 (54%)	0.45
IELSG score			
0–2	6/14 (43%)	8/17 (47%)	0.82
3–4	8/14 (57%)	9/17 (53%)	0.82
Pathologic type			
DLBCL	17/23 (74%)	18/24 (75%)	0.93
B-NHL (unclassified)	5/23 (22%)	5/24 (21%)	1.00
Unclear	1/23 (4%)	1/24 (4%)	1.00
Site of involvement			
Leptomeninges	2/23 (9%)	0/24 (0%)	0.23
Eyes	0/23 (0%)	3/24 (13%)	0.23
Nerve roots	0/23 (0%)	1/24 (4%)	1.00
Supplementary Treatment			
Systemic rituximab	8/23 (35%)	15/24 (63%)	0.06
Intrathecal chemotherapy	23/23 (100%)	21/24 (88%)	0.23
WBRT	9/23 (39%)	6/24 (25%)	0.30
ASCT	0/23 (0%)	2/24 (8%)	0.23
Courses < 3	2/23 (9%)	2/24 (8%)	1.00

*Median value (range). DLBCL, diffuse large B-cell lymphoma.

The total number of patients received intravenous rituximab was 8 (35%) in AB ± R alternative regimen group and 15 (63%) in the HD-MTX ± R group. 9 (39%) patients in AB ± R alternative regimen group and 6 (25%) patients in HD-MTX ± R regimen group received whole brain radiotherapy. 2 (8%) patients in HD-MTX ± R regimen group received autologous stem cell transplantation (ASCT). There were 2 patients who failed to finish 3 courses of chemotherapy due to early disease-related deaths or inadequate follow-up duration in each group.

### Survival data

**Figure 1:**
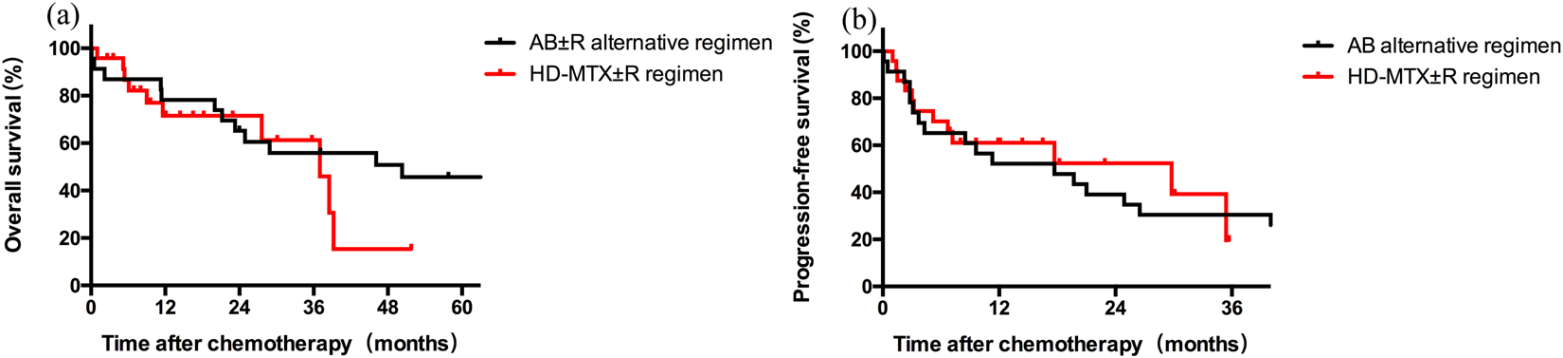
Overall survival and progression-free survival of patients with primary central nervous system lymphoma. (**a**) Overall survival. (**b**) Progression free survival.

Till the last follow-up, 14 patients died in AB ± R alternative regimen group, 9 of them were due to progressive disease. While 10 patients died in the HD-MTX ± R regimen group, 7 were due to progressive disease. There were 2 patients in AB ± R alternative regimen group and 1 patient in HD-MTX ± R regimen group died for rapid disease progression before the 2nd course chemotherapy (which was defined as early disease-related deaths). In consideration of the limited effects of the chemotherapy under this circumstances, we excluded the data of these 3 patients. The 2-year OS rate was 71.43% in AB ± R alternative regimen group and 74.62% in HD-MTX ± R regimen group respectively. And the 2-year PFS rate was 42.86% in AB ± R alternative regimen group and 54.64% in HD-MTX ± R regimen group respectively. No significant differences were found in OS (*p* = 0.20) or PFS (*p* = 0.98). The survival curve is shown as Fig. 1.

### Response to treatment

In AB ± R alternative regimen group, besides the early disease-related deaths, 2 more patients failed to be evaluated due to the patients’ personal denial. Among the rest patients who have finished evaluation, 7 (36.84%) patients achieved complete response within 6 months, and 6 (31.58%) patients achieved partial response within 6 months. In HD-MTX ± R regimen group, 2 patients were excluded due to their inadequate follow-up period (less than 6 months). Among the rest patients who have finished evaluation, 7 (33.33%) patients achieved complete response within 6 months, and 8 (38.10%) patients achieved partial response within 6 months. And the treatment response in both group was presented in Table 2 in detail.

**Table 2:** Treatment response of patients with primary central nervous system lymphoma at 6 months.

	AB ± R alternative regimen (n = 19)	HD-MTX ± R regimen (n = 21)	*p*-value
CR	7 (36.84%)	7 (33.33%)	1.00
PR	6 (31.58%)	8 (38.10%)	0.75
OR	13 (68.42%)	15 (71.43%)	1.00
SD	2 (10.53%)	2 (9.52%)	1.00
PD	4 (21.05%)	4 (19.05%)	1.00

CR, Complete Remission; PR, Partial Remission; OR, Overall Remission (defined as the rate of CR and PR); SD, Stable Disease; PD, Progressive Disease.

### Toxicity

In total, 142 cycles of AB ± R alternative regimen were administered, while 117 cycles of HD-MTX ± R regimen were administered. During these courses of induction chemotherapy, total 26 (18.31%) grade 3 or 4 toxicity events occurred to 9 (39.13%) patients in AB ± R alternative regimen, while total 21 (17.95%) grade 3 or 4 toxicity events occurred to 10 (41.67%) patients in HD-MTX ± R regimen group. No significant differences were found between the 2 groups (*p* = 1.00). The grade 3 or 4 hematologic toxicity rate was 7.04% in AB ± R alternative regimen and 5.98% in HD-MTX ± R regimen group respectively. No significant differences were found between the 2 groups (*p* = 0.80). The grade 3 or 4 neurologic toxicity rate was 4.23% in AB ± R alternative regimen and 1.71% in HD-MTX ± R regimen group respectively. No significant differences were found between the 2 groups (*p* = 0.30, seen in Supplementary Table 1).

### Subgroup analysis

**Figure 2:**
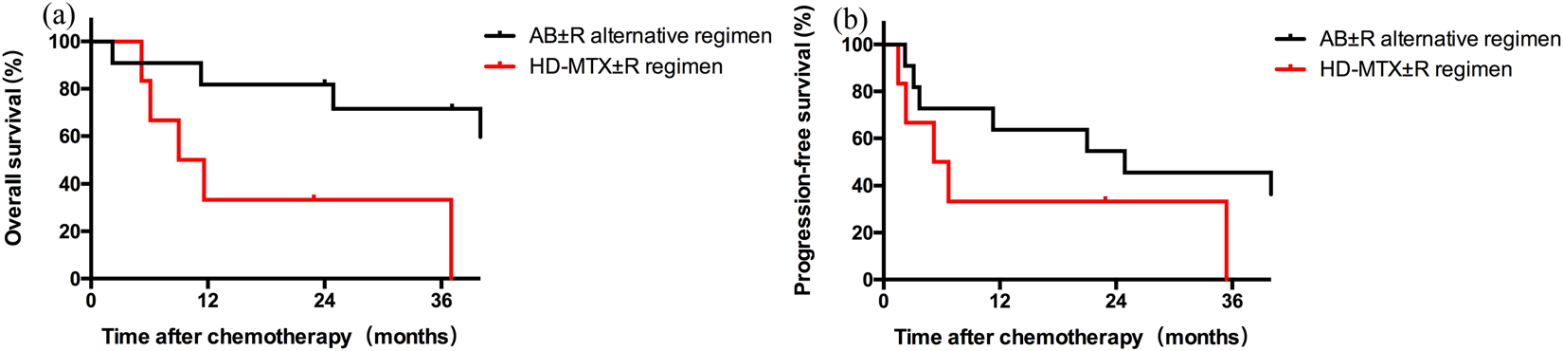
Subgroup analysis. (**a**) Overall survival of patients over 60 years old. (**b**) Progression free survival of patients over 60 years old.

In Age > 60 subgroup, 11 patients received AB ± R alternative regimen, 6 patients received HD-MTX ± R regimen. there is no difference between the two regimens in baseline characteristics and supplementary treatments except gender, (64% of patients in AB ± R alternative regimen group are male, 33% of patients in HD-MTX ± R regimen group were male, seen in Supplementary Table 2). The median OS and 2-year OS rate were 50.3 months and 81.82% in AB ± R alternative regimen group vs. 10.3 months and 33.33% in HD-MTX ± R regimen group, respectively, which is significant (p = 0.02). However, the difference of PFS curves showed no statistical significance with *p* = 0.15. The median PFS and 2-year PFS rate were 24.9 months and 54.55% in AB ± R alternative regimen group vs. 6.0 months and 33.33% in HD-MTX ± R regimen group, respectively. The survival curve in age over 60 subgroup was presented in Fig. 2.

In Age > 60 subgroup, 67 cycles of AB ± R alternative regimen were administered, while 27 cycles of HD-MTX ± R regimen were administered. No significant differences were found in grade 3 to 4 toxicity events rate (19.41% in AB ± R alternative regimen group vs. 22.22% in HD-MTX ± R regimen group, *p* = 0.78). The grade 3 or 4 hematologic toxicity rate was 10.45% in AB ± R alternative regimen and 7.4% in HD-MTX ± R regimen group respectively. No significant differences were found between the 2 groups (*p* = 1.00). The grade 3 or 4 neurologic toxicity rate was 2.99% in AB ± R alternative regimen and 3.70% in HD-MTX ± R regimen group respectively. No significant differences were found between the 2 groups (*p* = 1.00, seen in Supplementary Table 3).

In other subgroups based on age, serum LDH level, CSF protein level, deep lesions, ECOG score and IELSG score, we found no significant differences in OS, PFS, CR rate, or PR rate between the two regimens (seen in Supplementary Figure 1 and Supplementary Table 4). In addition, we compared the OS, PFS, CR rate, or PR rate between the patients who received rituximab or not in AB ± R alternative regimen group, as well as in HD-MTX ± R regimen group.

## Discussion

We performed a retrospective study of two different HD-MTX based combination chemotherapies that carried out in our center. Since AB ± R alternative regimen consist too many kinds of drugs, plus its complicated procedure and long duration of hospitalization, we changed the induction therapy into HD-MTX ± R regimen. There is no significant difference in survival and response rate between the two regimens, indicating the efficacy of HD-MTX ± R regimen was not inferior to that of AB ± R alternative regimen. The incidence of neurologic toxicity events of HD-MTX ± R group was slightly lower than that of AB ± R alternative regimen despite of no statistical significance, which may be due to the limited sample size. Also, there was no significant difference in grade 3–4 toxicity events between the two regimens either. Some tenser and more complicated systemic chemotherapy regimens, such as MBVP and CHOD/BVAM, consist similar components in our AB ± R alternative regimen, were reported with substantial toxicity^9,10^. Nadia N. Laack *et al*. reported 28% grade 3 or higher neurologic toxicity rate of CHOD/BVAM regimen^9^. Philip M.P. Poortmans *et al*. reported 10% toxic death rate of MBVP regimen^10^. Therefore, adverse effects, especially neurologic side effects, should be carefully monitored when tense and complicated chemotherapy used.

Some studies showed that, more complicated and tenser chemotherapy may not bring better treatment response or survival benefits^11^. The overall response rates of HD-MTX plus oral chemotherapy and more aggressive HD-MTX-based therapies were similar (73% vs. 75%), moreover, there was no difference between 2-year OS rates of HD-MTX plus oral chemotherapy and more aggressive HD-MTX-based therapies^12^. Some drugs in these more aggressive HD-MTX-based therapies may have poor blood-brain barrier penetration ability and questionable efficacy in PCNSL^11^. On the other hand, the intravenous administration of MTX was recommended at a dose of 3–8 g/m^2^, infused within 3 hours, with the interval less than 3 weeks^13^. Irrational administration of MTX compromise its effect in PCNSL. These points may explain the similar response and survival benefits in AB ± R alternative regimen and HD-MTX ± R regimen. Even AB ± R alternative regimen included more kinds of drugs, some of these drugs had poor blood-brain barrier penetration ability, and the interval of MTX in AB ± R alternative regimen was too long. While the clinical practice of MTX in HD-MTX ± R regimen was more rational.

Although the addition of rituximab were reported to be effective in PCNSL by some studies^14–16^, HD-MTX ± R regimen should be modified and improved to achieve better clinical outcome, since the blood-brain barrier penetration ability of rituximab is not satisfying enough^17^. Several other drugs were found to be effective in PCNSL, such as cytarabine, procarbazine, temozolomide, and thiotepa^16,18–20^. Addition of cytarabine to HD-MTX improved complete response rate (46% vs. 18%) and 3-year OS (46% vs. 32%)^18^. Another IELSG phase 2 trial showed that patients treated with methotrexate-cytarabine plus thiotepa and rituximab had higher complete remission rate than those treated with methotrexate-cytarabine alone (49% vs 23%)^16^. A randomised phase 2 trial in elderly population also presented satisfying response rates of methotrexate, procarbazine, vincristine, and cytarabine combination chemotherapy and methotrexate, temozolomide combination chemotherapy^20^. Further study about blood-brain barrier penetrated drugs are needed to discover the best HD-MTX based combination chemotherapy.

Age is an independent risk factor for PCNSL. Elderly patients have poorer prognosis than younger patients. In the Age > 60 subgroup, the overall survival of HD-MTX ± R regimen were obviously shorter than AB ± R alternative regimen. One reason we proposed to this result may be the inappropriate dose-reduction of MTX in elderly patients in HD-MTX ± R regimen, which is short of efficacy for controlling the progressive disease (50% of elderly patients in HD-MTX ± R regimen reduce MTX dose from 5 g/m^2^ to 3.5 g/m^2^, while age-based dose-reduction strategy was not adopted in AB ± R alternative regimen).

Concerning management approaches of PCNSL for elderly patients, it is widely believed that HD-MTX-based chemotherapy is safe and effective as initial treatment. However, the standard dose of intravenous MTX hasn’t reached a consensus. And the relationship between the dose of MTX and treatment response is not sure^21^ Some studies suggested that the dose-reduction of MTX might cause the decrease of drug concentration in cerebrum^22^, as well as the impairment of efficacy. However, the opposite side claimed that AUC_MTX_ was negatively correlated to overall survival^23^. So far, most studies adopted MTX dose-adjustment strategy in elderly patients with PCNSL^24^. Based on 5 prospective studies of adverse effects in elderly patients, the elderly population can well tolerate MTX at the dose lower than 3.5 g/m^2^ ^25–29^. If MTX is used at doses of 4–8 g/m^2^ as single agent, or at doses of 1.5–3.5 g/m^2^ in combination with other cytotoxic drugs, dose adjustment should be carefully carried out according to glomerular filtration rate^30^. Since lower creatinine clearance is independently associate with better prognosis^31^, the dose adjustment based on creatinine clearance does not affect treatment response and clinical outcome^32^. We believe that it is reasonable to adjust MTX doses according to creatinine clearance. Unfortunately, the dose-reduction in HD-MTX ± R regimen in our study was not strictly based on estimated glomerular filtration rate (eGFR), which might result in the disappointing outcome of these patients. We strongly recommend that, the dose adjustment of MTX should be carried out based on eGFR, instead of reducing doses for all elderly patients.

The elderly is a population more likely to experience adverse effects. AB ± R alternative regimen consist more kinds of drugs, which may result in various toxicity events. Another study found that CHOD/BVAM chemotherapy caused more obvious neurologic side effects in elderly patients^33^. In our study, we didn’t find significant difference of incidence of grade 3–4 toxicity events in Age > 60 subgroup between the two regimens, which perhaps due to limited sample size.

Our study was limited by the small sample size, especially in the subgroup analysis. AB ± R alternative regimen and HD-MTX ± R regimen were used in different periods. Such difference in treatment date and follow-up duration might result in many confounding factors, such as the supportive treatment and management abilities, which might influence the results.

In conclusion, the efficacy and safety of HD-MTX ± R regimen was not inferior to those of AB ± R alternative regimen. But HD-MTX ± R regimen showed unfavorable outcome in patients older than 60 years. We suggest to adjust this regimen by changing the dose-reduction strategy and adding other powerful drugs that can well penetrate blood-brain barrier. Further study about blood-brain barrier penetrated drugs are needed to discover the best HD-MTX based combination chemotherapy.

## Methods

### Patient selection

We performed a retrospective analysis of the newly diagnosed PCNSL patients accepted at least one course of AB ± R alternative regimen or single high-dose methotrexate ± rituximab (HD-MTX ± R) regimen at Peking Union Medical College Hospital, from August 1999 to August 2016. The inclusion criteria of this study are: the PCNSL diagnosis confirmed by pathologic examination, without any evidence of lymphoma outside brain, meninges, eyes, spinal cord or nerve roots detected by bone marrow biopsy, CT, or PET-CT. No prior chemotherapy was accepted except steroids. The exclusion criteria of this study includes infection of HIV or immunodeficiency caused by any other reasons; any other CNS disease including other type of tumor, hereditary disease, or metabolic disease; pregnancy or lactating.

The trial was approved by the Ethical Institutional Review Board of the Peking Union Medical College Hospital in accordance with the Declaration of Helsinki, and all patients gave written informed consent. The datasets generated during and/or analysed during the current study are available from the corresponding author on reasonable request.

### Data collected (BASELINE DATA)

We collected data of patient characteristics, patterns of care and outcome by reviewing medical records and making telephone calls.

#### Patient characteristics (basic patient information and baseline disease information)

Patients’ gender, age, date of the initial diagnosis, clinical classification, pathologic subtype, special site of involvement, results of cerebrospinal fluid examination, results of cranial MRI or PET-CT, ophthalmologic examination, Eastern Cooperative Oncology Group (ECOG) score, International Extranodal Lymphoma Study Group (IELSG) score, serum lactate dehydrogenase (LDH) level, and cerebrospinal fluid (CSF) protein level at first diagnosis, hemoglobin level, neutrophils counts, lymphocytes counts, platelet counts, ALT level, AST level, total bilirubin and direct bilirubin level within 14 days before the initiation of the first course of chemotherapy were all collected.

Approach to PCNSL management/care Pattern of care. 25 of 27 patients diagnosed with PCNSL between August 1999 and April 2012 were treated with AB ± R alternative regimen as induction therapy. Due to the complexity of its component and procedure, and long duration of hospitalization, we changed the induction therapy into HD-MTX ± R regimen from May 2012 to August 2016. The details of AB ± R alternative regimen were presented in another article from our center^34^. Briefly, Regimen A consist of methotrexate 3 g/m^2^·d, ifosfamide 800 mg/m^2^·d, vindesine 4 mg/d, dexamethasone 10 mg/m^2^·d. Regimen B consist of carmustine 125 mg/d, teniposide 50 mg/d, vindesine 4 mg/d, dexamethasone 10 mg/m^2^·d. The procedure of HD-MTX ± R regimen was similar to the regimen reported by James L. R, *et al*.^3^ with small difference of the dose of HD-MTX and the use of temozolomide, considering the adverse effects and patients’ tolerance. In HD-MTX ± R regimen group, Patients were given intravenous methotrexate 5 g/m^2^·d, over 4 hours, every 14 days. If the patients older than 65, the dose of MTX would be reduced 1/3. Whether rituximab (375 mg/m^2^·d) used or not is depended on patients’ preference and immune status.

In addition to systemic chemotherapy, all the patients without contraindications for lumbar puncture accepted intrathecal administration of cytarabine 50 mg, MTX 10 mg, and dexamethasone 5 mg during every course. Based on patients’ preference, some patients accepted WBRT as consolidation therapy. They were treated with 40–50 Gy WBRT plus a tumour-bed boost of 8–16 Gy.

All the patients received adequate hydration, urinary alkalization, and leucovorin rescue before and after methotrexate.

The date of initiation of chemotherapy, details of regimen, doses, courses of chemotherapy, and whether WBRT or ASCT received or not were all recorded. The adverse events and toxicity were evaluated according to *National Cancer Institute Common Toxicity Criteria for Adverse Events, version 3.0*.

#### Outcome

The follow-up of all patients were collected by reviewing medical records and making telephone calls. We recorded the treatments, responses, date and cause of death in the follow-up. The last follow-up was finished on November 1^st^, 2016. Responses to treatment were evaluated according to *The International Primary CNS Lymphoma Collaborative Group response criteria* ^35^, based on the results of cerebrospinal fluid examination, cranial MRI or PET-CT. Overall survival (OS) was calculated from the date of initiation of chemotherapy to the date of death from any cause, or censored if the subject was alive at the time of last follow-up. Progression-free survival (PFS) was calculated from the date of initiation of chemotherapy to the date the disease progressed, or censored if the patient had no progressive disease at the time of last follow-up.

#### Subgroup analysis

The subgroup analysis was based on age, serum LDH level, CSF protein level, deep lesions, ECOG score and IELSG score, which are independent prognostic indicator for PCNSL^2^, According to the prognostic value, the ECOG scores were grouped into 2 groups (0–1 vs > 1), while the IELSG scores were also grouped into 2 groups (0–2 vs 3–4), and the cutoff of LDH value and CSF protein level is 250 U/L and 0.45 g/L respectively. the OS and PFS were compared between the two chemotherapy in these subgroups.

### Statistics

Baseline characteristics of patients were summarized using descriptive statistics. We analyzed the difference in baseline characteristics and supplementary treatments between the two treatment regimen groups with Chi-square test or Fisher’s test. OS and PFS was estimated using the Kaplan-Meier method. The differences between two treatment regimen groups were evaluated with the log-rank test. The proportion of response to treatment was assumed to follow an independent binomial distribution. Chi-square test or Fisher’s test were used for proportional comparison. Two-tailed *p*-value ≤ 0.05 was considered to be statistically significant for all tests. All analyses were conducted using GraphPad Prism 6 (GraphPad Software, San Diego, CA, USA).

## Electronic supplementary material

Supplementary Materials
