# Supplementary material for: Efficacy and safety of HD-MTX based systemic chemotherapy regimens: retrospective study of induction therapy for primary central nervous system lymphoma in Chinese
Source: Sci Rep. 2017 Dec 6;7:17053. doi: 10.1038/s41598-017-17359-1 (PMC5719046; doi:10.1038/s41598-017-17359-1)
Supplement: Supplementary file 1 — Supplementary Materials [file 41598_2017_17359_MOESM1_ESM.pdf]

Efficacy and safety of HD-MTX based systemic chemotherapy regimens: retrospective study of induction therapy for primary central nervous system lymphoma in Chinese

Xiao Han[a1], Yali Ji[a1], Mingqi Ouyang[a], Tienan Zhu[a], Daobin Zhou[a]*

Supplementary table 1. Grade 3-4 toxicity of patients with primary central nervous system lymphoma.

	AB±R alternative regimen (n=142*)	MTX±R (n=117*)	p-value
Hematological toxicity	10（7.04%）	7（5.98%）	0.80
Neurologic toxicity	6（4.23%）	2（1.71%）	0.30
Infection	2（1.41%）	3（2.56%）	0.66
Hepatotoxicity	3（2.11%）	2（1.71%）	1.00
Electrolyte disorder	2（1.41%）	1（0.85%）	1.00
Cardiac toxicity	1（0.70%）	0（0.00%）	1.00
Aseptic necrosis of femoral head	1（0.70%）	0（0.00%）	1.00
Phlebitis	1（0.70%）	0（0.00%）	1.00
Thrombosis	0（0.00%）	2（1.71%）	0.20
Rectovaginal fistula	0（0.00%）	1（0.85%）	0.45
Renal toxicity	0（0.00%）	1（0.85%）	0.45
Diarrhea	0（0.00%）	1（0.85%）	0.45
Mucositis	0（0.00%）	1（0.85%）	0.45
Grade 3-4 toxicity events	26（18.31%）	21（17.95%）	1.00
Grade 3-4 toxicity patients	9/23*（39.13）	10/24*（41.67%）	1.00
Treatment-related deaths	1/23*（4.35%）	0/24*（0.00%）	0.49

*In AB±R alternative regimen group，23 patients received 142 cycles induction chemotherapy altogether; in MTX±R regimen group，24 patients received 117 cycles induction chemotherapy altogether.

Supplementary table 2. Characteristics of patients older than 60-year-old with primary central nervous system lymphoma.

	AB±R alternative regimen (n=11)	MTX±R (n=6)	p-value
Gender, male	7/11 (64%)	2/6 (33%)	0.03
ECOG performance status > 1	9/11 (82%)	4/6 (67%)	0.58
LDH > 250U/L	2/10 (20%)	1/6 (17%)	1.00
CSF protein > 0.45g/L	7/9 (78%)	4/4 (100%)	1.00
Deep lesion	4/10 (40%)	4/6 (67%)	0.61
IELSG score			
0-2	2/7 (29%)	0/4 (0%)	0.49
3-4	5/7 (71%)	4/4 (100%)	0.49
Pathologic type			
DLBCL	7/11 (64%)	6/6 (100%)	0.24
B-NHL (unclassified)	3/11 (27%)	0/6 (0%)	0.27
Unclear	1/11 (9%)	0/6 (0%)	1.00
Site of involvement			
Leptomeninges	1/11 (9%)	0/6 (0%)	1.00
Eyes	0/11 (0%)	0/6 (0%)	1.00
Nerve roots	0/11 (0%)	0/6 (0%)	1.00
Supplementary Treatment			
Systemic rituximab	4/11 (36%)	4/6 (67%)	0.33
Intrathecal chemotherapy	0/11 (0%)	0/6 (0%)	1.00
WBRT	5/11 (45%)	2/6 (33%)	1.00
ASCT	0/11 (0%)	1/6 (17%)	0.35

Supplementary table 3. Grade 3-4 toxicity of patients older than 60 years with primary central nervous system lymphoma.

	AB alternative regimen (n=67*)	MTX±R (n=27*)	p-value
Hematological toxicity	7（10.45%）	2（7.4%）	1.00
Neurologic toxicity	2（2.99%）	1（3.70%）	1.00
Hepatotoxicity	1（1.49%）	1（3.70%）	1.00
Electrolyte disorder	2（2.99%）	0（0.00%）	1.00
Cardiac toxicity	1（1.49%）	0（0.00%）	1.00
Infection	0（0.00%）	1（3.70%）	0.29
Thrombosis	0（0.00%）	1（3.70%）	0.29
Grade 3-4 toxicity events	13（19.41%）	6（22.22%）	0.78
Grade 3-4 toxicity patients	4/11*（36.36%）	3/6*（50.00%）	0.64
Treatment-related deaths	0/11*（0.00%）	0/6*（0.00%）	1.00

*In AB±R alternative regimen group，11 patients received 67 cycles induction chemotherapy altogether; in MTX±R regimen group，6 patients received 27 cycles induction chemotherapy altogether.

Supplementary table 4. Treatment response in subgroups.

SubGroup	Age > 60			ECOG≤1			LDH > 250U/L			CSF protein > 0.45g/L			With deep lesions			IELSG 3-4		
Regimen	AB±R alternative regimen (n=10)	MTX±R (n=6)	p-value	AB±R alternative regimen (n=5)	MTX±R (n=8)	p-value	AB±R alternative regimen (n=3)	MTX±R (n=3)	p-value	AB±R alternative regimen (n=9)	MTX±R (n=12)	p-value	AB±R alternative regimen (n=7)	MTX±R (n=13)	p-value	AB±R alternative regimen (n=5)	MTX±R (n=9)	p-value
CR	4 (40.0%)	1 (16.7%)	0.59	3 (60.0%)	4 (50.0%)	1.00	1 (33.3%)	0 (0.0%)	1.00	4 (44.4%)	3 (25.0%)	0.40	3 (42.9%)	4 (30.8%)	0.65	2 (40.0%)	1 (11.1%)	0.51
PR	3 (30.0%)	3 (50.0%)	0.61	1 (20.0%)	1 (12.5%)	1.00	0 (0.0%)	0 (0.0%)	1.00	3 (33.3%)	5 (41.7%)	1.00	2 (28.6%)	5 (38.5%)	1.00	1 (20.0%)	4 (44.4%)	0.58
OR	7 (70.0%)	4 (66.7%)	1.00	4 (80.0%)	5 (62.5%)	1.00	1 (33.3%)	0 (0.0%)	1.00	7 (77.7%)	8 (66.7%)	0.66	5 (71.4%)	9 (69.2%)	1.00	3 (60.0%)	5 (55.6%)	1.00
SD	1 (10.0%)	0 (0.0%)	1.00	0 (0.0%)	1 (12.5%)	1.00	0 (0.0%)	1 (33.3%)	1.00	1 (11.1%)	1 (8.3%)	1.00	1 (14.3%)	1 (7.7%)	1.00	1 (20.0%)	1 (11.1%)	1.00
PD	2 (20.0%)	2 (33.3%)	0.60	1 (20.0%)	2 (25.0%)	1.00	2 (66.7%)	2 (66.7%)	1.00	1 (11.1%)	3 (25.0%)	0.60	1 (14.3%)	3 (23.1%)	1.00	1 (20.0%)	3 (33.3%)	1.00

SubGroup	Age≤60			ECOG > 1			LDH≤250U/L			CSF protein≤0.45g/L			No deep lesions			IELSG 0-2		
Regimen	AB±R alternative regimen (n=9)	MTX±R (n=15)	p-value	AB±R alternative regimen (n=14)	MTX±R (n=13)	p-value	AB±R alternative regimen (n=11)	MTX±R (n=16)	p-value	AB±R alternative regimen (n=4)	MTX±R (n=3)	p-value	AB±R alternative regimen (n=10)	MTX±R (n=8)	p-value	AB±R alternative regimen (n=5)	MTX±R (n=6)	p-value
CR	3 (33.3%)	6 (40.0%)	0.68	4 (28.6%)	3 (23.1%)	1.00	4 (36.4%)	5 (31.3%)	1.00	2 (50.0%)	0 (0.0%)	0.43	4 (40.0%)	3 (37.5%)	1.00	3 (60.0%)	2 (33.3%)	0.57
PR	3 (33.3%)	5 (33.3%)	0.70	5 (35.7%)	7 (53.8%)	0.45	4 (36.4%)	8 (50.0%)	0.70	1 (25.0%)	2 (66.7%)	0.49	3 (30.0%)	3 (37.5%)	1.00	1 (20.0%)	3 (50.0%)	0.55
OR	6 (66.7%)	11 (73.3%)	1.00	9 (64.3%)	10 (76.9%)	0.68	8 (72.7%)	13 (81.3%)	0.66	3 (75.0%)	2 (66.7%)	1.00	7 (70.0%)	6 (75.0%)	1.00	4 (80.0%)	5 (83.3%)	1.00
SD	1 (11.1%)	2 (13.3%)	1.00	2 (14.3%)	1 (7.7%)	1.00	1 (9.1%)	1 (6.3%)	1.00	0 (0.0%)	1 (33.3%)	0.43	1 (10.0%)	1 (12.5%)	1.00	0 (0.0%)	1 (16.7%)	1.00
PD	2 (22.2%)	2 (13.3%)	0.61	3 (21.4%)	2 (15.4%)	1.00	2 (18.2%)	2 (12.5%)	1.00	1 (25.0%)	0 (0.0%)	1.00	2 (20.0%)	1 (12.5%)	1.00	1 (20.0%)	0 (0.0%)	1.00

Supplementary figure 1. Overall survival and progression-free survival in subgroups.

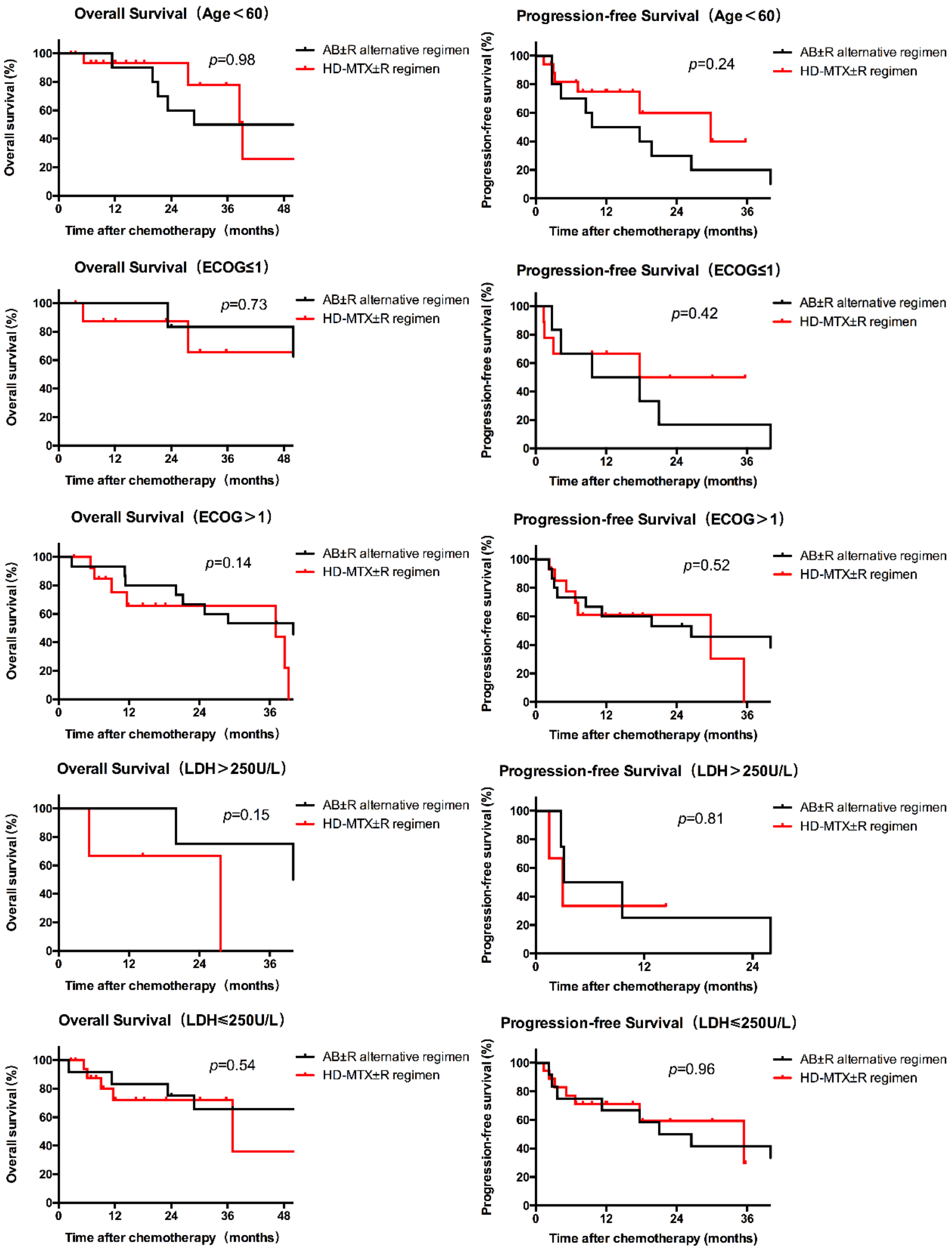

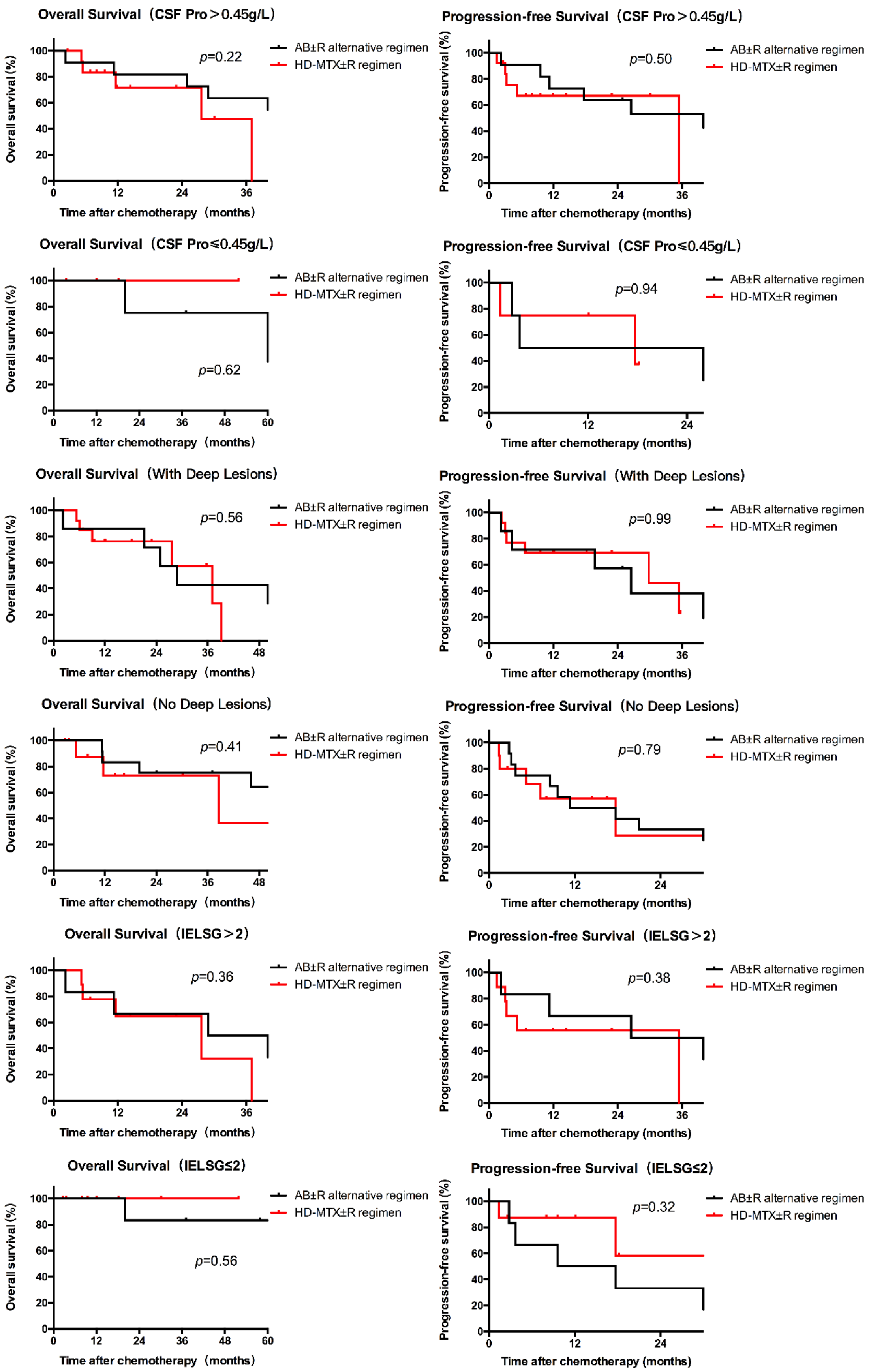
Overall Survival（CSF Pro＞0.45g/L）
Overall survival (%)
Time after chemotherapy（months）
p=0.22
AB±R alternative regimen
HD-MTX±R regimen
Progression-free Survival（CSF Pro＞0.45g/L）
Progression-free survival (%)
Time after chemotherapy (months)
p=0.50
AB±R alternative regimen
HD-MTX±R regimen
Overall Survival（CSF Pro≤0.45g/L）
Overall survival (%)
Time after chemotherapy（months）
p=0.62
AB±R alternative regimen
HD-MTX±R regimen
Progression-free Survival（CSF Pro≤0.45g/L）
Progression-free survival (%)
Time after chemotherapy (months)
p=0.94
AB±R alternative regimen
HD-MTX±R regimen
Overall Survival（With Deep Lesions）
Overall survival (%)
Time after chemotherapy（months）
p=0.56
AB±R alternative regimen
HD-MTX±R regimen
Progression-free Survival（With Deep Lesions）
Progression-free survival (%)
Time after chemotherapy (months)
p=0.99
AB±R alternative regimen
HD-MTX±R regimen
Overall Survival（No Deep Lesions）
Overall survival (%)
Time after chemotherapy（months）
p=0.41
AB±R alternative regimen
HD-MTX±R regimen
Progression-free Survival（No Deep Lesions）
Progression-free survival (%)
Time after chemotherapy (months)
p=0.79
AB±R alternative regimen
HD-MTX±R regimen
Overall Survival（IELSG＞2）
Overall survival (%)
Time after chemotherapy（months）
p=0.36
AB±R alternative regimen
HD-MTX±R regimen
Progression-free Survival（IELSG＞2）
Progression-free survival (%)
Time after chemotherapy (months)
p=0.38
AB±R alternative regimen
HD-MTX±R regimen
Overall Survival（IELSG≤2）
Overall survival (%)
Time after chemotherapy（months）
p=0.56
AB±R alternative regimen
HD-MTX±R regimen
Progression-free Survival（IELSG≤2）
Progression-free survival (%)
Time after chemotherapy (months)
p=0.32
AB±R alternative regimen
HD-MTX±R regimen

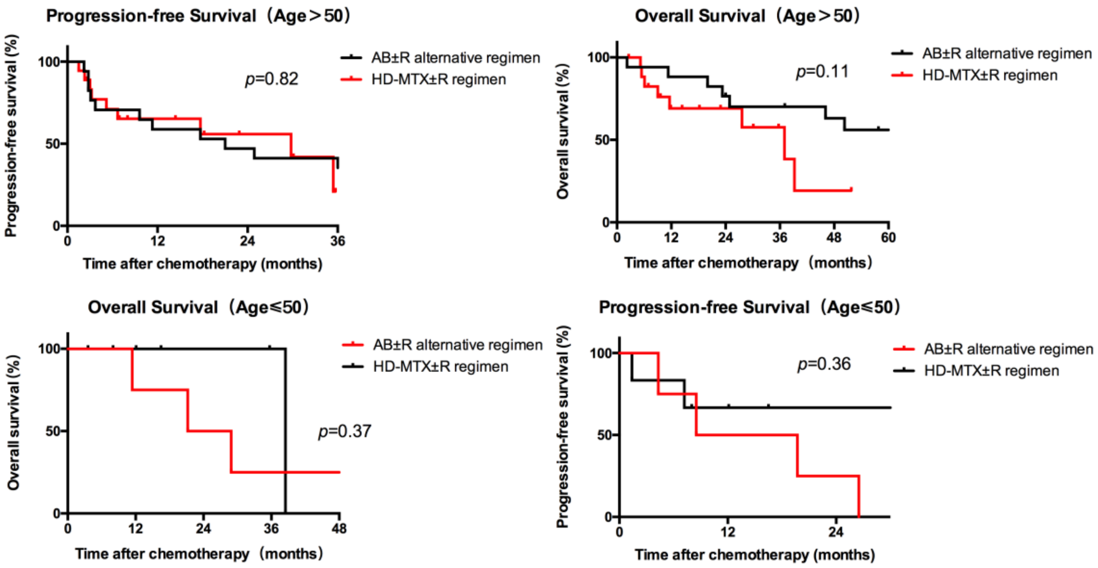

Overall survival and progression-free survival in subgroups of Age<60, ECOG>1, ECOG≤1, serum LDH elevated, serum LDH normal, CSF protein elevated, CSF protein normal, with deep lesions, without deep lesions, IELSG 0-2, IELSG 3-5, separately. Updated with subgroup analysis for patients with age older or younger than 50 years old.
